# Thromboangiitis Obliterans: Changing Demographics for a Preventable Disease

**DOI:** 10.7759/cureus.3869

**Published:** 2019-01-11

**Authors:** Ryan Stefancik

**Affiliations:** 1 Osteopathy, University of Pikeville-Kentucky College of Osteopathic Medicine, Pikeville, USA

**Keywords:** thromboangiitis obliterans, buerger's disease, smoking, smoking cessation

## Abstract

Thromboangiitis obliterans (TAO), otherwise known as Buerger's Disease, is a rare, small-vessel vasculitis strongly associated with cigarette smoking, that when left untreated can cause vessel destruction and necrosis of the distal extremities leading to amputation. The patient being presented is a 46-year-old Caucasian female who has been smoking since the age of fifteen and shows characteristics of TAO on angiography. The uniqueness of this case lies in the epidemiology; the typical TAO patient is a 20 to 40-year-old Asian male. However, over the last few decades, the typical patient population for TAO has been shifting. Our patient represents this changing demographics of TAO patients that include a greater percentage of women, non-Asian ethnicities, and the elderly. This patient represents an opportunity to follow the disease progression and learn more about the pathophysiology of TAO as it pertains to its shifting demographics.

## Introduction

Buerger’s Disease, also known as Thromboangiitis obliterans (TAO), is a rare, small-vessel vasculitis affecting primarily the arteries of the distal extremities. This vasculitis is a segmental, non-atherosclerotic disease that damages the vessels and nerves in the hands and feet, that if untreated will eventually lead to gangrene and potential amputations. The pathology behind this vessel damage is thought to be constant thrombosing and recanalization, with the rate of recanalization decreasing over time leading to occluded vessels that are eventually “obliterated”, thereby giving the disease it’s namesake [1]. 

The typical TAO patient is a 20 to 40-year-old Asian male with a history of cigarette smoking. However, as smoking has decreased in popularity in the United States, researchers believe the demographics for the disease are shifting. Our case represents this possible shifting patient population, seeing as how our patient is a 46-year-old Caucasian female. This patient is still in the beginning phases of the disease, in that it has not progressed to extremity necrosis. We hope this case sheds more light on a shifting group of patients for a disease that can have severe morbidity implications.

## Case presentation

A 46-year-old Caucasian female with a significant past medical history for cervical cancer, anticardiolipin antibody syndrome, peripheral artery disease, hyperlipidemia, anxiety, and depression presented to the Medical Center in Bowling Green, Kentucky due to a recent ultrasound that showed elevated pressures of a femoropopliteal bypass graft in her right lower extremity. On angiography of her right lower extremity, she was found to have high-grade stenosis in the upper portions of her bypass along with a diseased popliteal artery with tandem stenotic lesions. The patient then underwent an AngioJet thrombectomy of the right femoropopliteal bypass which began thrombosing immediately afterwards.

The patient was admitted to the hospital for post-operative recovery and the on-call internal medicine physician took the patient onto their service, at which point a complete history was taken and a physical exam was performed. The patient was found to be morbidly obese (body mass index 42.5), well developed, well-nourished patient in no acute distress, with a normal respiratory and cardiac exam. The patient was found to have tenderness along the anterior of the right lower extremity, and mild inflammation along the lateral portion of the left foot. The left lower extremity pulse was slightly weaker than the right.

**Figure 1:**
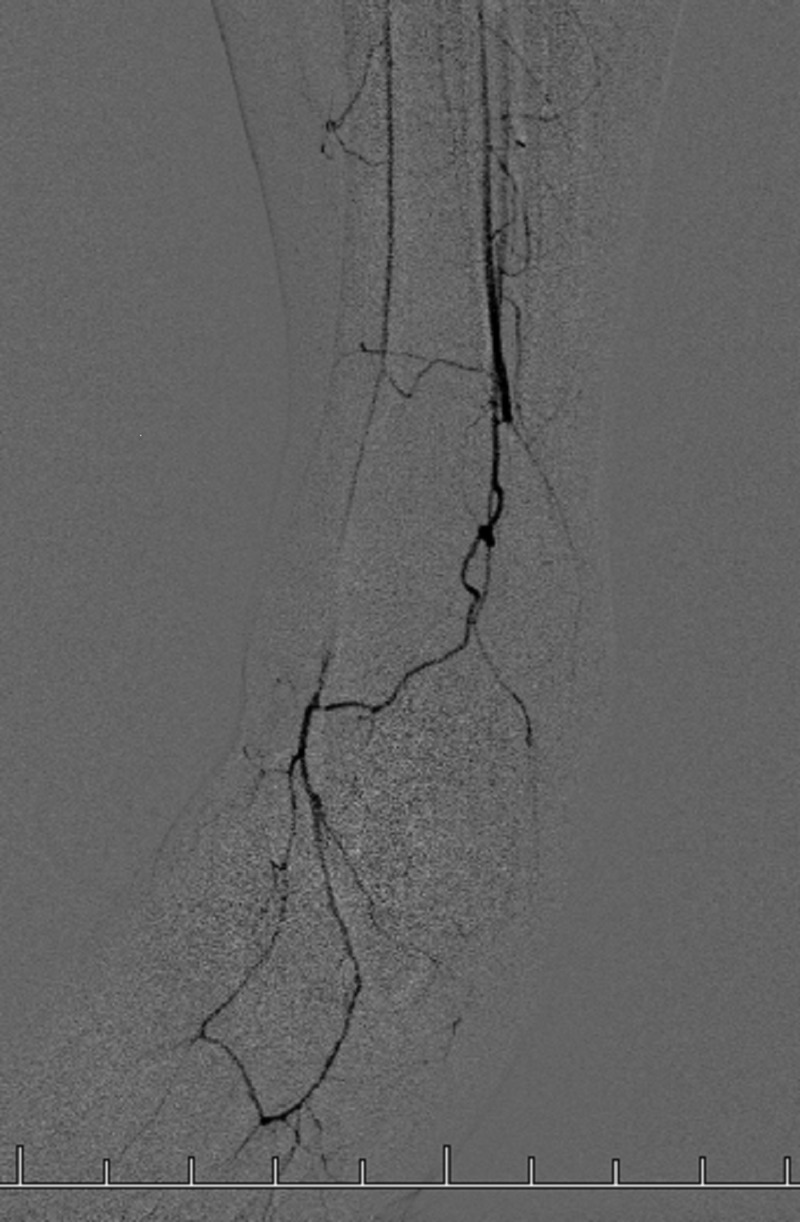
Left Lower Extremity Angiography Angiography of the left ankle and foot shows the tortuous path and corkscrew turning of the posterior tibial artery and subsequent medial plantar artery; a pattern typically seen in TAO patients.

**Figure 2:**
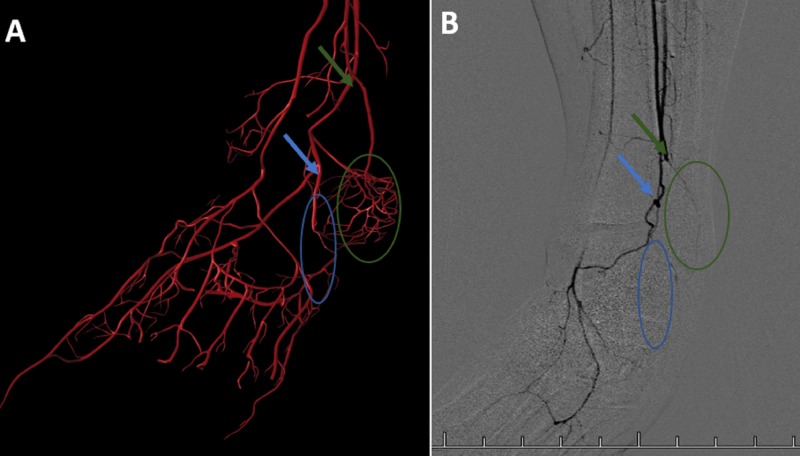
Left Lower Extremity Angiography Compared to Computer-generated Vasculature Image A is the computer-generated vasculature of a healthy left foot. Image B is an angiogram of the left foot of our patient. The blue arrow represent the branching point of the lateral plantar artery coming off of the posterior tibial artery. The blue circle represents the typical course of the lateral plantar artery. The green arrow represents the typical location where the posterior tibial artery branches into tributaries for the calcaneal arterial anastomosis. The green circle represents the typical location of the calcaneal arterial anastomosis. Image B shows the obliteration of the lateral plantar artery and calcaneal arterial anastomosis, along with the occlusion of the proximal portions of these arterial tributaries.

A lower extremity angiography was performed on this patient prior to the AngioJet thrombectomy which revealed damage to the distal left lower extremity caused by the patient’s TAO. The posterior tibial artery is shown to have developed a tortuous, corkscrew-like path through the left foot, an anatomical feature associated with TAO (Figure 1). Vessels contributing to the left calcaneal anastamosis along with the lateral plantary artery were shown to be obliterated from this patient’s TAO (Figure 2). The angiography also shows arterial occlusions where the calcaneal anastamosis vessels and lateral plantar artery typically branch off of the posterior tibial artery (Figure 2).

The patient currently works from a home office. She claims to have smoked a pack to a pack and a half of cigarettes daily since her early teens, giving her a thirty to fifty pack-year history. The patient admits to consuming alcohol three to four times per month. The patient denies any illicit drug use.

## Discussion

Since the 1990’s there has been an increase in the percentages of women and the elderly that have developed TAO. Historically, most data about the progression of the disease has been through case studies on the typical 20 to 40-year-old Asian male patient [2]. As the demographics continue to include a greater portion of women and individuals over 40 years of age, it is important to track the disease in these patients. One major reason is the rare signs and symptoms of TAO may give us more insight into the pathophysiology of the disease that is more commonly reported in these historically unique patient populations but not limited to central retinal artery occlusion and migratory thrombophlebitis [3-4].

The first physical signs of TAO occurred when the patient presented to the hospital in February of 2018 claiming right leg claudication for the previous three weeks. During this visit, the first toe of the right foot was noted to be pale and unhealthy around the nailbed without ulceration. The physical exam findings during that initial February visit, the 8/16/18 hospital stay, and the angiography done on the same date, in addition to the patient’s medical history, support the diagnosis of TAO.

The patient is still in the beginning stages of TAO, in that she has yet to show any signs of Raynaud’s phenomenon or color changes in her distal extremities (Table 1). However, the disease process has begun as shown by obliteration of the left lateral plantar artery and left calcaneal arterial anastomosis, as well as the tortuous medial plantar artery. While further follow-ups for her other complex peripheral vascular disorders will most likely require future procedures, for now, she is on her way towards halting the disease via smoking cessation. Unfortunately, smoking cessation is the only proven way to halt disease progression, with no definitive treatment being available. Currently, there are studies being conducted to evaluate the efficacy of new drugs and certain procedures dependent on disease staging; surgical sympathectomies, mononuclear cell therapy, iloprost, etc. [5-7]. Despite these promising studies, for now, we are only able to offer our patients information and means of smoking cessation.

**Table 1:** Thromboangiitis Obliterans Typical Case HLA: Human leukocyte antigen

Thromboangiitis Obliterans	
Risk Factors	smoking, male, hypercoaguable state, 20-40yo, Asian, HLA specific
Symptoms	Claudication with walking, Raynaud's phenomenon, discoloration of distal extremities
Labs	Doppler: decreased flow to distal extremities. Angiogram/Arteriograph: obstruction/obliteration of distal small arteries, tortuous and corkscrew pattern seen in distal vessels
DDx	Autoimmune disorders, diabetes, vasculitis, arterial emboli
Treatment	Initial: smoking cessation, possibly anticoagulants. Late-phase: sympathectomy, amputation of necrosed digits/limb segments

This case also highlights a problem with electronic medical record keeping and the laziness that can go along with it. In 2013, this patient was diagnosed with possible Buerger’s disease, and the physician subsequently documented this in the electronic medical records system as Berger's Disease, otherwise known as IgA nephropathy. Unfortunately, this patient has never had any renal disorders, but was victim to a miskeying of her true diagnosis of Buerger’s disease aka TAO. From that initial incorrect documentation of this patient’s disorder in 2013 until 8/16/18, TAO was never discussed with the patient. Up until her most recent visit, the patient had always denied smoking cessation assistance including varenicline, bupropion, and nicotine patches. After a long meaningful discussion with her physician, the patient was adamant on receiving a nicotine patch prescription and during her follow-up visit in 10/18, she had been compliant with the patches, having not smoked since she had been educated about her disease in August.

## Conclusions

This case represents an opportunity to expand our knowledge of an uncommon vasculitis in a traditionally uncommon patient population. Following patients with TAO that fall into the patient populations that are becoming more commonplace (female, greater than 40 years of age at onset, non-Asian ethnicity) will hopefully offer more insight into the disease pathophysiology as well as more effective management plans and methods of treatments.
